# Kava (*Piper methysticum*) consumption patterns and conceptualizations: results from an online survey

**DOI:** 10.1186/s13011-026-00728-3

**Published:** 2026-05-05

**Authors:** Katherine Hill, Cianna Piercey, Hollis C. Karoly, Kirsten E. Smith

**Affiliations:** 1 Department of Epidemiology of Microbial Diseases, Yale School of Public Health, New Haven, CT USA; 2 Department of Psychology, Colorado State University, Fort Collins, CO USA; 3 Department of Psychiatry, School of Medicine, University of Colorado Anschutz Medical Campus, Aurora, CO USA; 4 Department of Psychiatry and Behavioral Sciences, Johns Hopkins University School of Medicine, Baltimore, MD USA

**Keywords:** Kava, *Piper methysticum*, Kratom, Harm-reduction, Alcohol

## Abstract

**Background:**

‘Kava,’ or ‘kava kava,’ (*Piper methysticum*) is a psychoactive plant indigenous to the Pacific Islands. Historical consumption is reported to provide anxiolytic and sedating effects. In regions where kava is native, it has been used in religious and cultural practices, as well as for medicinal purposes. The mechanisms by which kavalactones – the best studied psychoactive constituent of kava – may mediate pharmacological effects include enhancing the GABA_A_ receptor, blocking of sodium and calcium channels, blocking reuptake of norepinephrine dopamine, and inhibiting MAO-B. In the United States, products containing kava have proliferated in recent years alongside the rise of ethnobotanical tea bars serving kava preparations.

**Methods:**

Between December–July 2024, an anonymous online survey on legal psychoactive products was conducted using convenience sampling. Eligible individuals had to be *≥* 18 years old and endorse lifetime use of kratom, kava, tianeptine, and/or akuamma seed.

**Results:**

Of the 368 participants, almost half (*n* = 180, 48.9%) had used kava during their lifetime. Those who reported lifetime use of kava were younger on average (*p* = 0.001), more racially diverse (*p* = 0.041), and had a higher proportion of being employed or in school (*p* = 0.012) than those where did not use kava in their lifetime. Kava was purchased online (40.6%) or in ethnobotanical tea bars (31.7%). Many reported infrequent consumption (average = 4.4 months/year and 9.1 days/30 days). Lifetime use of alcohol, kratom, and cannabis was common. Approximately one-third of participants had consumed kava more than 100 times, with commonly reported perceived effects from kava experienced in the minutes and hours after consumption included reduced general anxiety (33.7%), improved mood (32.9%), reduced social anxiety (25.5%), and sedation (23.4%). Most respondents had favorable conceptualizations of kava, with only 11.7% characterizing that kava alone as habit-forming. Many (51.7%) conceptualized kava as an alcohol replacement with 18.3% and 16.7% using kava as a short- and/or long-term substitute for alcohol, respectively.

**Conclusions:**

Kava use practices are diverse, though perceived kava effects appear mostly mild. The proliferation and diversification of kava products pre-mixed with other psychoactive botanicals requires study as the benefit-risk profile may change. Clinicians should be aware of kava use for harm-reduction and other purposes.

**Supplementary Information:**

The online version contains supplementary material available at 10.1186/s13011-026-00728-3.

## Background

‘Kava,’ or ‘kava kava,’ (*Piper methysticum*) is a psychoactive plant endemic to the Pacific Islands [1]. The indigenous use of kava, typically through mastication or as a beverage wherein the root is macerated into water or coconut water, is reported to provide anxiolytic and sedating effects [2–4]. In regions where kava is native, it has been used in religious and cultural practices, as well as medicinally [5]. While kava root is believed to have been consumed for centuries, documentation on kava use began with colonization and was recognized in Western medicine in the late 1880’s as a potential anxiolytic [1, 6].

Kavalactones are among the best studied chemical constituents of kava, with six of approximately 18 kavalactones demonstrating pharmacological activity; kavain and methysticin are typically found in the highest quantities while dihydrokawain, dihydromethysticin, yangonin, and desmethoxyyangonin are found in smaller quantities [3, 7, 8]. Minor constituents include amino acids, minerals (potassium, sodium), and flavokavins (A, B and C) [9]. Although the exact mechanisms of kava’s effects in humans are not perfectly elucidated some putative mechanisms by which kavalactones may mediate pharmacological effects include GABA_A_ receptor-enhancing mechanisms, blockade of sodium and calcium channels, as well as blockade of reuptake of norepinephrine and dopamine, and MAO-B inhibition [9–12]. Kavain and dihydrokavain in particular are associated with reported anxiolytic activity [12–14]. In clinical studies examining kava’s anxiolytic effects between 1989 and 2004, daily doses of kavalactones administered as part of the investigation ranged from 60 to 240 mg, with the longest duration of use 210 mg of kavalactones for 24 weeks [15, 16]. Across these studies, the average kavalactones per day was approximately 158 mg.

Some adverse effects have been reported or observed – both indigenously and more recently in the United States – taking kava for prolonged periods or large amounts. This includes hepatotoxicity and dermopathy, with the former appearing most frequently [2, 17–20]. Although there is a record of historical use for kava in regions where it is indigenous, such use does not translate to the United States where kava preparations are relatively novel and often consumed alongside myriad other psychoactive substances [14, 21–24].

In recent years, kava products have proliferated in the United States. Some kava products contain low amounts of kavalactones are sold as dietary supplements typically in capsule form at health and wellness stores or high-end groceries and commercial tea bags may advertise kava as an relaxing constituent [25]. Parallel to this, products containing concentrated amounts of kava root extract at doses that are high relative to commercial dietary supplements containing kava sold in health and wellness stores are being sold online and in convenience stores in liquid shots or tonics, sometimes blended with other psychoactive botanicals (e.g., CBD, kratom) [26, 27]. Kava is also prepared into drinks at ethnobotanical tea bars selling kava and kratom [1, 6, 28–30].

Kava is not controlled or scheduled under the Controlled Substances Act by the Drug Enforcement Administration and may be considered by the U.S. Food and Drug Administration (FDA) to be and “old dietary ingredient,” meaning that kava could be sold in certain forms as a dietary supplement [31, 32]. Both FDA and the Centers for Disease Control and Prevention have released reports and consumer advisories, warning those who consume kava of the potential adverse effects, namely those related to liver injury and FDA has not evaluated efficacy for any reported kava effects [28, 33, 34]. Products containing kava marketed as a dietary ingredient or supplement are expected to have a reasonable basis of safety for their indicated use. Despite the increased presence of kava in regions where it is not indigenous, its use remains underexplored.

Accordingly, we sought to characterize the attributes of adults with a history of kava use. Among this group we aimed to identify kava consumption behaviors, other substances consumed contemporaneous to kava, use motivations, and conceptualizations of kava. We also explored how kava use is experienced across settings, including ethnobotanical tea bars that serve kava and other psychoactive botanical-derived beverages (e.g., kratom), and to better understand general perceptions and attitudes of kava use. Other findings from this project related to substance use disorder for kava are presented elsewhere [24].

## Methods

Between December 2023 and July 2024, we conducted a cross-sectional study among a convenience sample adults exploring the use of legal products with varying psychoactive characteristics that are commonly sold in gas stations/online stores and often touted to be alternative means of self-management for various medical conditions, recreation, and more. To be eligible, participants had to be *≥* 18 years of age and to endorse lifetime use of one of the following substances: kratom, kava, akuamma seed, and/or tianeptine. Participants were recruited using digital flyers and social media through platforms such as Reddit and X. Prior to accessing the survey, all interested candidates were provided an information sheet which contained study aims and procedures, including that the survey was not compensated, that participation was voluntary, and that the data would be used for research purposes. Data were collected on Qualtrics along with IP addresses only for the purposes of avoiding duplicate responses; validity checks were included in the survey, and quality checks from Qualtrics used to identify potential bots. The Johns Hopkins University School of Medicine Institutional Review Board determined this did not qualify as human subjects’ research due to the anonymous study design. Requests for data may be made to the corresponding author.

### Survey and data analysis

The survey was administered online using Qualtrics (Qualtrics, Provo, UT). The survey instrument was developed from prior kratom surveys with additional items adapted for assessment of kava [35]. The full survey is available in supplemental materials. Broadly, the survey consisted of questions pertaining to sociodemographic characteristics, including age in years, race/ethnicity (which was dichotomized for analysis to White/Caucasian and Other Race/Ethnicity due to small cell sizes for racial/ethnicity minority groups), gender/sex, employment, education, relationship status, incarceration history, income, status of having children, substance use disorder (SUD) history, recovery status, and zip code. Zip codes were then used to assign participants to a geographic location using Rural-Urban Community Area Codes [36].

Survey respondents who reported lifetime kava use were then asked questions that allowed us to quantify their kava use behaviors, including frequency of use, effects from use, purchasing history, typical dosage, and knowledge and conceptualizations of kava. Open-text items were used to query about the settings in which participants typically consume kava and which aspects they ‘most’ and ‘least’ enjoy about using kava in a bar setting. Additionally, participants were asked “Is there anything else you would like to tell us about your experiences using kava?” Due to its confidential nature, the Johns Hopkins University School of Medicine Institutional Review Board determined this did not qualify as human subjects’ research.

Continuous variables were summarized using means and standard deviations; categorical variables were summarized using frequencies and percentages. T-tests for continuous variables and chi-square tests for categorical variables were used to examine distributions of sociodemographic variables comparing participants reporting lifetime kava use to those without lifetime kava use. Survey responses from open-text items were independently coded by two researchers (CJP and HCK) and analyzed in order to identify reoccurring themes. Specifically, CJP and HCK independently reviewed responses to each question and generated an initial codebook. They then jointly refined and finalized codes prior to engagement in independent focused coding. Following independent coding, they jointly resolved divergence in codes and generated themes.

## Results

### Sociodemographic characteristics

Of the total sample (*N* = 368), almost half had used kava in their lifetime (*n* = 180, 48.9%) while *n* = 188 had no lifetime kava use experience (though they may have experience(s) with other substances). Table 1 shows the sociodemographic characteristics of the sample, stratified by lifetime kava use. Those who reported lifetime use of kava were younger on average (38.0 vs. 42.2, *p* = 0.001), more racially diverse (*p* = 0.041), and had a higher proportion working or in school (*p* = 0.012). Fewer in this group had children compared to respondents who had never tried kava (*p* = 0.025). Relatively equal proportions of those with and without lifetime use of kava considered themselves to be in SUD recovery (*p* = 0.996) and had been previously diagnosed with any SUD for alcohol and/or other drugs (*p* = 0.854).

**Table 1:** Sociodemographic characteristics for those with a lifetime use of kava compared to those without a lifetime use of kava

Characteristic	Lifetime Use of Kava (*n* = 180)		No Lifetime Use of Kava (*n* = 188)		*p*-value*
	*N* or Mean	% or SD	*N* or Mean	% or SD	
**Age**	38.03	11.21	42.22	13.01	**0.001**
**Race/Ethnicity**					**0.041**
White/Caucasian	156	86.67	175	93.09	
Other Race/Ethnicity ^a^	24	13.33	13	6.91	
**Gender/Sex**					0.993
Male	111	36.67	115	37.23	
Female	66	61.67	70	61.17	
Non-binary	3	1.67	3	1.60	
**Employment**					**0.012**
Working full-time, working part-time, or a student	148	82.22	134	71.28	
Disabled	19	10.56	19	10.11	
Unemployed	8	4.44	18	9.57	
Retired	5	2.78	17	9.04	
**Education**					0.288
Finished High School / GED	15	8.33	20	10.64	
Some College	36	20.00	40	21.28	
College Degree or Higher ^b^	127	70.56	121	64.36	
Other	2	1.11	7	3.72	
**Relationship Status ** ^c^					0.336
In Relationship	112	62.22	126	67.02	
Not in Relationship	68	37.78	62	32.98	
**Incarceration History**					0.748
Yes	28	15.56	27	14.36	
No	152	84.44	161	85.64	
**Income**					0.400
Less than $30,000	38	21.11	53	28.19	
$30,000 to less than $60,000	34	18.89	37	19.68	
$60,000 to less than $90,000	34	18.89	32	17.02	
Over $90,000	74	41.11	66	35.11	
**Children**					**0.025**
Yes	71	39.44	96	51.06	
No	109	60.56	92	48.94	
**Geographic Location ** ^d^					0.610
Urban	86	50.59	75	45.18	
Suburban	54	31.76	58	34.94	
Rural	30	17.65	33	19.88	
**Considers Self in Recovery**	46	25.56	48	25.53	0.996
**Ever Diagnosed with Substance Use Disorder**	47	26.11	54	28.72	0.854

* p-value represents Chi Square test for categorical variables and t-test for continuous variables

^a^ Other Race/Ethnicity includes: Hispanic, Native American, including Native Alaskan or Pacific Islander, Biracial, Middle-Eastern, Asian, African American/Black, and/or Other Race/Ethnicity

^b^ College Degree or Higher includes: Associates/Vocational Degree, Bachelor’s Degree, Master’s Degree, PhD, JD, MD, and/or DO

^c^ In Relationship includes: Currently Married, or In Committed Partner Relationship (monogamous or polyamorous). Not in relationship includes: Single and looking for a long-term partner, Single and looking only for short-term hook-ups and sex, Divorced, or Widowed

^d^ Based on Rural-Urban Commuting Area (RUCA) Codes. Participants provided their zip code, which were used to find corresponding RUCA code. A score of 1, 4, or 7 were categorized as urban; 2, 5, 8 were categorized as suburban; and 3, 6, 9, or 10 were categorized as rural. There are 33 missing values (*n* = 170 for kava, *n* = 166 for no kava)

### Kava use patterns and other substance use history

On average, participants were 30.9 years old when first tried kava (Table 2). Supplemental Fig. 1 shows that most respondents began using kava within the last decade (2014–2024). Participants reported consuming kava relatively infrequently, reporting use 4.4 months per year on average (SD = 4.5) and 9.1 days per 30 days on average (SD = 11.0). Approximately one-third of kava consumers had taken kava more than 100 times. As shown in Supplemental Table 1, those who used kava also had an extensive history of other substance use, including other psychoactive botanical products such as kratom (82.2%), cannabis (77.8%), and CBD (72.2%), and of prescription medications, including opioids (62.8%), anti-depressants (57.8%), and anxiolytic medications (43.3%). Most (92.2%) had a history of alcohol use.

**Table 2:** Kava use patterns among those with lifetime use of kava (*n* = 180)

	*N* or Mean	% or SD
**Age of First Use**	30.94	11.07
**Number of Months Used in the Past Year**	4.38	4.48
**Number of Days Used in the Past 30 Days**	9.10	11.01
**Has Used More Than 100 Times**	58	32.22
**Effects Felt Minutes and Hours After Taking Kava**		
Reduced general anxiety	124	33.70
Improved mood	121	32.88
Reduced social anxiety	94	25.54
Sedation	86	23.37
Sleepiness	48	13.04
Pain relief	47	12.77
Euphoria	46	12.50
Reduced craving for another drug	32	8.70
Increased energy	23	6.25
Loss of appetite	22	5.98
Increased focus	20	5.43
Increased productivity	15	4.08
Increased alertness	13	3.53
Slower heart rate	13	3.53
Increased libido/sex drive	11	2.99
Slower breathing/respiration	8	2.17
Foggy memory	8	2.17
Reduced kava craving	5	1.36
Desire to eat	4	1.09
Other	23	6.25
**Where Purchased Kava ** ^a^		
Online US-based vendor that I used previously and regularly	73	40.56
Kava bar	57	31.67
Online US-based vendor that I used previously but irregularly	40	22.22
Herbal/vitamin shop	23	12.78
Smoke shop	16	8.89
High-end natural products store	15	8.33
Kratom bar	12	6.67
From a friend	9	5.00
Online vendor from unknown location	8	4.44
Head shop	7	3.89
Online vendor directly based outside of the US	7	3.89
Gas station/convenience store	5	2.78
From a store selling multiple products not otherwise classified.	5	2.78
Other	20	11.11
**How Often You Typically Changed Where Purchased Kava**		
Very often or Often	6	3.33
Occasionally	31	17.22
Not often or Never	131	72.78
It depended on other circumstances	12	6.67
**Kava Knowledge**		
Extremely knowledgeable	33	18.33
Pretty knowledgeable	56	31.11
Somewhat knowledgeable	66	36.67
Not at all knowledgeable	23	12.78
Unsure	2	1.11
**Been to Kava Bar or Kava/Kratom Bar**		
Yes	75	41.67
**Plan to Keep Using**		
Yes, I plan to keep using the same amount	76	42.22
Yes, I plan to increase my kava use	6	3.33
Yes, but I plan to decrease me kava use	6	3.33
No, I plan to stop using kava altogether	9	5.00
Unsure	13	7.22
I don’t have plans about kava either way	70	38.89

^a^ Participants could select multiple options, so columns may not sum to total frequency or 100%

Most purchased kava products online (40.56%) or in a kava bar (31.67%). Respondents reported feeling the following effects from kava minutes and hours after consumption: reduced general anxiety (*n* = 124, 33.7%), improved mood (*n* = 121, 32.9%), reduced social anxiety (*n* = 94, 25.5%), and sedation (*n* = 86, 23.4%). All other effects were endorsed by less than 15% of participants on average. In open-text responses, some respondents reported that the effects of kava were mild, minimal, or in some cases, “not worth the effort.” Several respondents shared that they prefer to use kava in combination with other substances to potentiate kava’s effects. For example, one person stated, “by itself, kava is alright at best but when taken with other substances like phenibut, tianeptine, kratom, and THC, it opens it up.”

Regarding the setting in which kava was typically consumed, open-text responses indicated that many respondents consumed kava at home. However, consuming kava within a bar setting or a non-bar social setting (e.g., party) was also common. Some also reported consuming kava at work or school, or prior to entering these settings. When respondents were asked what they ‘most’ and ‘least’ enjoy about using kava in a bar setting, there were a diversity of responses (Supplemental Table 2). The social atmosphere of the bars was seen as a positive aspect of kava consumption within this setting; for instance, one participant explained “The kava bar feels like a community center, usually supporting local charities and cares about the community around them.” Participants also enjoyed the convenience of prepared drinks, the taste and variety of products to choose from, the ability to try new products, and communal sharing of knowledge about kava. However, these same reasons that attracted some to the bars made others disinterested. Some respondents indicated kava bars were “cliquey” or “crowded” whereas others disliked the “cost and not being sure of the source of the kava,” which included concerns about “unclear dosing.”

### Kava dosing

With respect to dosing information (Table 3), just over half of respondents reported not consuming kava daily (*n* = 92, 51.1%). When taken daily, less than a quarter of participants reported taking kava two or more times per day (*n* = 39, 21.7%). More than a third of participants indicated that they did not consume kava weekly (*n* = 67, 37.2%), yet those who did use weekly consumed somewhere between 1 and 6 servings weekly (*n* = 75, 41.7%).

**Table 3:** Kava dose information among those with lifetime use of kava

Characteristic	Lifetime Use of Kava (*n* = 180)	
	*N* or Mean	% or SD
**Servings Typically Taken Per Day**		
0	92	51.11
1	48	26.67
2	17	9.44
3	9	5.00
4+	13	7.22
**Servings Typically Taken Per Week**		
0	67	37.22
1–6	75	41.67
7–13	12	6.67
14–20	13	7.22
21+	13	7.22
**Kava Dosing Units Typically Use ** ^a^		
Cups of tea/juice/kava drink preparation	51	28.33
Grams	47	26.11
Tablespoons	34	18.89
Extract	21	11.67
Liquid Ounces	20	11.11
Teaspoons	18	10.00
Spoonfuls	17	9.44
Capsules (standard size)	9	5.00
Edibles	5	2.78
Capsules (jumbo size)	2	1.11
Other	15	8.33
**Most Frequent Kava Dosing Unit**		
Cups of tea/juice/kava drink preparation	43	23.89
Grams	37	20.56
Tablespoons	31	17.22
Spoonfuls	14	7.78
Liquid Ounces	14	7.78
Teaspoons	13	7.22
Extract	10	5.56
Capsules (standard size)	6	3.33
Edibles	2	1.11
Capsules (jumbo size)	1	0.56
Other	9	5.00
**Typical Dose of Kava**		
Cups of tea/juice/kava drink preparation (*n* = 51)	2.16	1.35
Grams (*n* = 47)	21.54	72.59
Tablespoons (*n* = 34)	2.88	1.79
Spoonfuls (*n* = 17)	2.53	1.97
Liquid Ounces (*n* = 20)	6.45	6.37
Teaspoons (*n* = 18)	2.06	1.39
Extract (*n* = 21)	8.75	33.75
Capsules (standard size) (*n* = 9)	2.56	1.59
Edibles (*n* = 5)	1.60	0.89
Capsules (jumbo size) (*n* = 2)	2.50	2.12
**Average Time Takes to Feel Effects from Kava Dose (** ***n*** ** = 158)**		
Seconds	12	7.59
Minutes	142	89.87
Hours	4	2.53
**Average Time Takes to Stop Feeling Effects from Kava Dose**		
Minutes	27	15
Hours	143	79.44
Unsure (would take more before the effects wear off)	10	5.56
**Felt Effects Every Time Use Kava**		
Yes, I feel an effect every time (or almost every time)	121	67.22
No, I never (or rarely) feel an effect	25	13.89
Neither of those is quite true	34	18.89
**How Often Changed Kava Dosing Routine**		
Very often or Often	26	14.44
Occasionally	29	16.11
Not often or Never	107	59.44
It depended on other circumstances	18	10.00
**Since First Used Kava**,** Dosing/Intake Has**		
Never took it regularly enough for there to be much change	54	30.00
Held steady and not changed	44	24.44
I have completely stopped using kava	30	16.67
Decreased	25	13.89
Increased	20	11.11
Other	7	3.89

^a^ Participants could select multiple options, so columns may not sum to total frequency or 100%

Most commonly, respondents reported dosing kava in units of cups for drink preparations (*n* = 51, 28.3%), grams (*n* = 47, 26.1%), and tablespoons (*n* = 34, 18.9%), with a corresponding average dose of 2.2 cups (SD = 1.4), 21.5 g (SD = 72.6), and 2.9 tablespoons (SD = 1.8). From the doses/servings the participants prepared, most participants indicated that it typically took just ‘minutes’ to feel kava’s embodied effects (n = 142, 89.9%) with effects lasting hours (n = 143, 79.4%). Most indicated feeling these effects every time or almost every time they used kava (n = 121, 67.2%) with a majority also not increasing their intake over time.

### Kava conceptualizations

Table 4 provides an overview of how participants with lifetime use of kava perceive the botanical; for instance, corresponding with the primary effects felt by respondents, 71.1% endorsed kava as “relaxing” (*n* = 128) and 66.1% endorsed kava as “therapeutic” (*n* = 119). Most participants indicated that kava “should be legal” (*n* = 114, 63.3%) and was “helpful” (*n* = 103, 57.2%). Over half of the sample conceptualized kava as an “alcohol replacement” (*n* = 93, 51.7%) and many respondents used open-text responses to emphasize their use kava for harm-reduction purposes, reporting use of kava as a substitute for alcohol and other substances (e.g., prescription medications). One participant said, “I believe kava saved my life from alcoholism” while another stated “It is a great alcohol replacement, and it makes it easy when I’m around people who drink a lot.” In the survey, very few participants thought kava was “addictive” (*n* = 5, 2.8%) or “problematic” (*n* = 6, 3.3%). This was mirrored by open-text responses, where many participants reported a belief that kava was “safe” or “safer” than other substances they had previously tried. However, in the survey, over 10% of respondents conceptualized kava as “habit-forming” (*n* = 21, 11.7%).

**Table 4:** Conceptualizations of kava use reported by subset of those with kava use in their lifetime

Characteristic	Lifetime Use of Kava (*n* = 180)	
	*N*	%
**Conceptualization of Kava ** ^a^		
Relaxing	128	71.11
Therapeutic	119	66.11
Should be legal	114	63.33
Helpful	103	57.22
Alcohol replacement	93	51.67
Is not nearly as strong as alcohol	92	51.11
Is not nearly as strong as opioids	86	47.48
Medicinal	78	43.33
Targeted by government agencies such as the FDA	59	32.78
Not very potent	57	31.67
Sedating	56	31.11
A real benefit to my daily life	55	30.56
Stigmatized	55	30.56
Isn’t an opioid	54	30.00
Isn’t a benzodiazepine	52	28.89
Overhyped	42	23.33
A combination of stimulating and sedating	42	23.33
Isn’t regulated across vendors who sell kava	32	17.78
Targeted by DEA for criminalization	32	17.78
Media panic	28	15.56
Homeopathic	28	15.56
Too expensive	26	14.44
Naturopathic	24	13.33
Inconsistent in its effects	24	13.33
Habit-forming	21	11.67
Lifesaving	21	11.67
Stimulating	17	9.44
Boring	16	8.89
Study drug	13	7.22
Sometimes adulterated with other products	12	6.67
Potent	8	4.44
Pre-workout	7	3.89
Problematic	6	3.33
Addictive	5	2.78
Energy shot	4	2.22
Is a benzodiazepine	3	1.67
Is an opioid	1	0.56
An increasing problem or burden for me	1	0.56
**Conceptualizations Changed Since First Heard of Kava**		
Yes	49	29.88
No	115	70.12
**Conceptualizations Changed to More or Less Favorable**		
More favorable	35	71.43
Less favorable	7	14.29
It’s too complicated to say	5	10.20
Other	2	4.08

^a^ Participants could select multiple options, so columns may not sum to total frequency or 100%

### Kava use motivations

As shown in Supplemental Table 3, kava use motivations were wide-ranging, though the most commonly endorsed motivations included use for recreation, fun or to relax (*n* = 108, 60.0%), to self-treat anxiety symptoms (*n* = 101, 56.1%), to reduce social anxiety (*n* = 87, 48.3%), to feel less bad in general and improve quality of life (*n* = 75, 41.7%), and because they viewed kava as safer than other substances (*n* = 75, 41.7%). With respect to kava use related to harm-reduction, 18.3% (*n* = 33) and 16.7% (*n* = 30) reported using kava as a short- and/or long-term substitute or replacement for alcohol, respectively. Other participants using kava as a short-term substitute or replacement for benzodiazepines (*n* = 19, 10.56%).

## Discussion

To our knowledge, this is one of the first surveys to explore kava experiences and use patterns outside of regions where it is indigenous. Our survey found that kava consumers were younger and more racially diverse on average than consumers of other legal psychoactive substances (i.e., kratom, akuamma seed, tianeptine) included in this survey sample. Among those who had ever tried kava, just under half used kava daily with approximately 22.0% using more than one serving of kava per day. While there are few self-report data in the literature on kava use behaviors, effects, or conceptualizations, several of our findings comport with other self-report from adults with a history of kava use who have posted on social media about their use patterns, including contemporaneous use with other psychoactive botanicals [14]. It is unclear the extent to which kava was concurrently used in real-time with other substances, including kratom, which is often sold pre-mixed with kava or is served alongside kava in ethnobotanical tea bars [29]. Similar to many of the open text items and conceptualizations, kava’s effects have previously been described as predominantly mild [14]. Indeed, some posting to social media about kava have discussed ways for potentiating kava’s effects or, conversely, using kava to potentiate the effects from another substance [14].

### Reported kava use for anxiety, relaxation, and recreation

Approximately one third of respondents reported that, within the minutes and hours after kava consumption, kava produced feelings of generalized anxiety relief, while a quarter endorsed kava as reducing social anxiety. Consonant with these effects, approximately 75% also conceptualized kava as “relaxing” with more than half of respondents indicating that they were motivated to use kava due to its perceived ability to provide relaxation and self-treatment for anxiety symptoms. Such findings mirror previous work by Pont-Fernandez et al. (2023), who found that Reddit posts related to kava involved a wide range of use motivations, including kava use to achieve an altered state or intoxication but also use for the purposes of relaxation or as an anxiolytic in the form of self-medication [14]. Although reducing unwanted states, such as anxiety, were common, the most commonly reported motivation included using kava for “recreation, fun, or to relax” within the same survey item. As such, more work is needed to examine the use of kava for each, although we suspect that the lines between these may not always be clear and are certainly not mutually exclusive use motivations.

### Reported kava use for alcohol harm-reduction

Almost 95% of those with lifetime kava use also had a reported lifetime use of alcohol and nearly half conceptualized kava as an alcohol replacement. Fewer (< 20%) kava consumers reported that they were using kava as either a short- or long-term substitute or replacement for alcohol. Taken together, though, findings provide some support for the idea that kava is used as a form of harm-reduction for alcohol. Among these respondents, motivations for using kava as a form of harm-reduction did not extend to benzodiazepines or opioids. As approximately 25% of lifetime kava consumers considered themselves to be in recovery during the time of survey completion, it is possible that some of these individuals were engaged in non-abstinent recovery practices, as have been documented elsewhere with both kava and kratom [29, 35]. We anticipate that in addition to surveys and ambulatory methods for data collection, qualitative work will be required in order to develop a better initial understanding of kava use practices among populations for which historical use data do not exist and for which kava is being used as a replacement for other substances [21, 37].

### Limitations

This exploratory study was limited in several respects, including the fact that it was cross-sectional and comprised of a small convenience sample. Thus, findings should not be interpreted as representative of the broader population of adult kava consumers nor should findings be used to draw conclusions related to temporality or causality. Because kava has a long history of traditional use, some respondents expressed (i.e., in their open text responses) a desire for the survey items to have permitted greater cultural consideration in terms of contrasting traditional use practices with consumption largely based in the United States. Indeed some respondents noted in open text field that they believed that this survey should have allowed for more culturally driven perspectives from what indigenous kava use might look like compared to that occurring primarily in the mainland U.S. Such considerations should be undertaken in future work on kava. Though it is evident, we believe it is worth underscoring that kava use among our respondents, who mostly consumed kava in U.S.-based ethnobotanical tea bars or through purchasing pre-made kava products (e.g., seltzers, blended extracts, shots) online, should in no way be confused with historical kava consumption practices. Such practices have largely – but not always – been ceremonial, cultural, medicinal, and infrequent (i.e., not daily) and include important variations across cultures within the many regions were kava is indigenous and over time [38–42]. Lastly, we also focused on mostly normative or neutral aspects of kava use. Elsewhere, we present findings of SUD for kava and adverse effects.

## Conclusions

In regions where kava is indigenous, it has been mostly used in both normative and nonproblematic ways, largely in keeping with historical and cultural practices, but also has in recent years become a substance of concern even in indigenous regions with the dependence liability not fully elucidated [43–45]. We suspect that as use grows in regions where kava has no historical use, such as the U.S., that consumption practices will be varied and, like many other legal psychoactive substances, will be adopted by consumers for a variety of reasons that may change over time. As sales of kava products proliferate and include co-formulation with other psychoactive ingredients significantly more research is needed to better understand kava products. U.S.-based clinicians should also be aware of kava use and asses for possible drug interactions or effects that may develop from chronic daily consumption (e.g., dependence, liver injury) as part of a holistic patient-centered care approach.

## Supplementary Information

Below is the link to the electronic supplementary material.

Supplementary Material 1

Supplementary Material 2

Supplementary Material 3

Supplementary Material 4

## Data Availability

Requests for data may be made to the corresponding author.
