# Supplementary figures and images for: Kava (Piper methysticum) consumption patterns and conceptualizations: results from an online survey
Source: Subst Abuse Treat Prev Policy. 2026 May 5;21:43. doi: 10.1186/s13011-026-00728-3 (PMC13317249; doi:10.1186/s13011-026-00728-3)

**Supplemental Figure 1. Distribution of When Participants (n=180) First Used Kava**

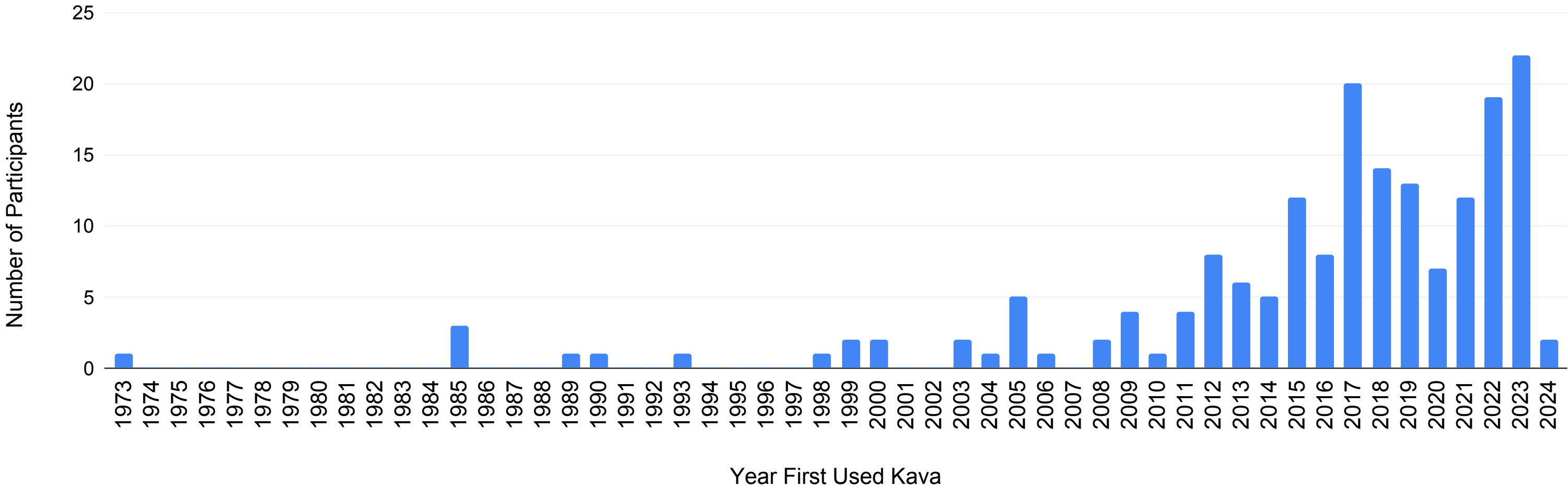

Supplement: Supplementary file 1 — Supplementary Material 1 [file 13011_2026_728_MOESM1_ESM.pdf]
