# Supplementary material for: Kava (Piper methysticum) consumption patterns and conceptualizations: results from an online survey
Source: Subst Abuse Treat Prev Policy. 2026 May 5;21:43. doi: 10.1186/s13011-026-00728-3 (PMC13317249; doi:10.1186/s13011-026-00728-3)
Supplement: Supplementary file 2 — Supplementary Material 2 [file 13011_2026_728_MOESM2_ESM.docx]

| **Supplemental Table 1. Lifetime substance use^a^ for participants reporting lifetime kava use** | | | |
| --- | --- | --- | --- |
|  | **Lifetime *Kava* Use**  **(n=180)** | |  |
| **Substance** | n | % |  |
| Caffeine | 178 | 98.89 |  |
| Alcohol | 166 | 92.22 |  |
| Kratom | 148 | 82.22 |  |
| Cannabis (not prescribed) | 140 | 77.78 |  |
| Cannabidiol (CBD) | 130 | 72.22 |  |
| Combustible tobacco (e.g., cigarettes, cigars, hookah) | 127 | 70.56 |  |
| Prescription opioids (prescribed) | 113 | 62.78 |  |
| Anti-depressants (prescribed) | 104 | 57.78 |  |
| E-cigarettes/vapes | 103 | 57.22 |  |
| Delta 8 THC | 90 | 50.00 |  |
| Hallucinogens (e.g., LSD, mushrooms) | 89 | 49.44 |  |
| Delta 9 THC | 78 | 43.33 |  |
| Anti-anxiety drugs (prescribed) | 78 | 43.33 |  |
| Powder cocaine | 66 | 36.67 |  |
| Prescription opioids (not prescribed) | 65 | 36.11 |  |
| Anti-anxiety drugs (not prescribed) | 65 | 36.11 |  |
| Ecstasy/MDMA | 65 | 36.11 |  |
| Amphetamines (not prescribed) | 59 | 32.78 |  |
| Phenibut | 45 | 25.00 |  |
| Amphetamines (prescribed) | 38 | 21.11 |  |
| DXM/dextromethorphan | 37 | 20.56 |  |
| Marijuana (prescribed) | 36 | 20.00 |  |
| Anti-psychotics (prescribed) | 32 | 17.78 |  |
| Ketamine | 29 | 16.11 |  |
| Methamphetamine | 26 | 14.44 |  |
| DMT | 26 | 14.44 |  |
| Modafinil/Provigil (not prescribed) | 23 | 12.78 |  |
| Akuamma Seed | 22 | 12.22 |  |
| Racetams | 22 | 12.22 |  |
| Heroin | 21 | 11.67 |  |
| Crack/rock/freebase cocaine | 18 | 10.00 |  |
| Synthetic marijuana | 16 | 8.89 |  |
| Suboxone/Subutex (not prescribed) | 14 | 7.78 |  |
| Fentanyl | 12 | 6.67 |  |
| Suboxone/Subutex (prescribed) | 9 | 5.00 |  |
| Methadone (not prescribed) | 9 | 5.00 |  |
| Tianeptine Sodium | 9 | 5.00 |  |
| Tianeptine Sulphate | 8 | 4.44 |  |
| Anti-depressants (not prescribed) | 7 | 3.89 |  |
| Bath salts | 5 | 2.78 |  |
| Anti-psychotics (not prescribed) | 5 | 2.78 |  |
| Methadone (prescribed) | 4 | 2.22 |  |
| Other | 15 | 8.33 |  |
| ᵃ Participants could select multiple answer choices, so frequencies will be larger than total, and percentages will sum to over 100%. | | | |
|  |  | | |
