# Supplementary material for: Kava (Piper methysticum) consumption patterns and conceptualizations: results from an online survey
Source: Subst Abuse Treat Prev Policy. 2026 May 5;21:43. doi: 10.1186/s13011-026-00728-3 (PMC13317249; doi:10.1186/s13011-026-00728-3)
Supplement: Supplementary file 3 — Supplementary Material 3 [file 13011_2026_728_MOESM3_ESM.docx]

| **Supplemental Table 3. Motivations for kava use^a^ among participants reporting lifetime kava use** | | | |  | |
| --- | --- | --- | --- | --- | --- |
|  | **Lifetime *Kava* Use**  **(n=180)** | | |  |  |
| **Motivation** | n | % |  |  |  |
| For recreation, fun, or to relax | 108 | 60.00 |  |  |  |
| To self-treat anxiety symptoms | 101 | 56.11 |  |  |  |
| To reduce social anxiety | 87 | 48.33 |  |  |  |
| To feel less crappy in general and improve your quality of life | 75 | 41.67 |  |  |  |
| Because kava is safer than other substances | 75 | 41.67 |  |  |  |
| As a sleep aid | 69 | 38.33 |  |  |  |
| To self-treat depression symptoms | 41 | 22.78 |  |  |  |
| As a short-term substitute/replacement for alcohol | 33 | 18.33 |  |  |  |
| To achieve a euphoric high | 31 | 17.22 |  |  |  |
| As a long-term substitute/replacement for alcohol | 30 | 16.67 |  |  |  |
| To self-treat short-term pain (i.e., acute pain management) | 20 | 11.11 |  |  |  |
| To self-treat post-traumatic stress symptoms | 19 | 10.56 |  |  |  |
| To self-treat long-term pain (i.e., chronic pain management) | 19 | 10.56 |  |  |  |
| As a short-term substitute/replacement for benzodiazepines | 19 | 10.56 |  |  |  |
| Because you prefer the kava “high” to “highs” from other drugs | 17 | 9.44 |  |  |  |
| To take as a part of a self-designed “stack” of other drugs that help you feel good | 16 | 8.89 |  |  |  |
| To address occasional feelings of sleepiness or low energy | 16 | 8.89 |  |  |  |
| Doctors will not prescribe the drugs you need | 15 | 8.33 |  |  |  |
| Because could not get a hold of other, more preferred drugs | 10 | 5.56 |  |  |  |
| As a long-term substitute/replacement for benzodiazepines | 9 | 5.00 |  |  |  |
| As a long-term substitute/replacement for opioids | 8 | 4.44 |  |  |  |
| To relieve withdrawal symptoms from alcohol | 6 | 3.33 |  |  |  |
| To relieve withdrawal symptoms from a variety of different drugs | 5 | 2.78 |  |  |  |
| To self-treat bipolar symptoms | 3 | 1.67 |  |  |  |
| To relieve withdrawal symptoms from non-prescribed opioids or heroin | 3 | 1.67 |  |  |  |
| Other | 24 | 13.33 |  |  |  |
| ᵃ Participants could select multiple answer choices, so frequencies will be larger than total, and percentages will sum to over 100%. | | |  |  |  |
