# Supplementary material for: Kava (Piper methysticum) consumption patterns and conceptualizations: results from an online survey
Source: Subst Abuse Treat Prev Policy. 2026 May 5;21:43. doi: 10.1186/s13011-026-00728-3 (PMC13317249; doi:10.1186/s13011-026-00728-3)
Supplement: Supplementary file 4 — Supplementary Material 4 [file 13011_2026_728_MOESM4_ESM.docx]

**Supplemental Table 2. Summary of Open-text Findings Related to Kava Use Experiences**

| **Open-text Item** | **N** | **Summary of Qualitative Findings** |
| --- | --- | --- |
| Please describe the setting in which you typically consume kava.  N=180 | 180 | - Physical settings   - Home (N=121)   - Bar (N=37)   - Non-bar social setting (e.g., party) (N=18)   - Work or school (or prior to entering these settings) (N=12)   - Running errands (N=1)   - Outside or in nature (N=2) - Other contextual descriptions   - Before or after exercise (N=3)   - Before sleep (N=11) or upon waking (N=1)   - After work (N=7)   - Before meditation, ritual, or ceremony (N=2) |
| What do you most enjoy about using kava in a bar setting? | 75 | - Socializing and building community (N=44) - Appreciation for the ambiance and atmosphere of a bar setting (N=25), particularly being able to enjoy this type of setting without alcohol being served (N=9) - Enjoyment of products (N=10), specifically, not having to prepare one’s own drinks, enjoying the taste and variety of products to choose from, and being able to try new products - Sharing of knowledge about kava (N=4), specifically learning more about kava and how it’s prepared from bartenders - Use in a bar setting is fun (N=2) - Supervised consumption (i.e., others are around if unwanted or adverse effects arise) (N=1) |
| What do you least enjoy about using kava in a bar setting? | 75 | - Disliking of bar atmosphere (e.g., overcrowding, not having anyone to go to bars with) (N=23) - Products served at the bars (N=7)   - Concerns about the quality and sourcing of products and the manner in which products are prepared   - Unclear dosing of products, lack of knowledge about the products being served, or disliking the fact that kratom is also served at the bars - Lack of convenience, having to drive home from the bar/ being responsible about consumption (N=3) - Consumption in a bar not aligned with personal use motives (N=2) - Cost (N=19) |
| Is there anything else you would like to tell us about your experiences using kava? | 152 | - Use of kava for harm reduction purposes and as an alternative for alcohol, prescription medications, or other substances (N=22) - Perceived safety of kava   - N=19 commented on the safety of kava, stating that kava was “safe” or “safer” than other substances, that kava does not have the unwanted effects of other substances (e.g. hangover), that kava is not addictive or habit-forming, and that they don’t experience problems related to use   - N=1 said kava was “very addictive”   - N=1 acknowledged that kava has “cons” but didn’t specify further - Use of kava for relaxation or self-treatment of anxiety, depression, or other mental health symptoms (N=14) - Report that the effects of kava are mild or minimal (N=10) - Disliking of kava’s effects, belief that kava is not worth the effort of preparing, or not a preferred substance (N=9) - Discussion of community and social aspects of kava use (N=7) - Experiences consuming kava with other substances (N=5) - Perception of kava as a net-benefit or “life changing” (N=5) - Kava Advocacy   - N=3 advocated greater public education on kava   - N=4 advocated to keep kava legal   - N=4 advocated for more research on kava - Infrequent or minimal use of kava (N=14) - Survey feedback (N=5), particularly the need for culturally driven perspectives within kava research - Other responses   - N=2 said they use kava for sleep   - N=1 noted importance of sourcing   - N=1 reported spiritual use   - N=1 said kava suppresses their appetite   - N=1 said kava sparked interest in other “nootropics” |
